# Integration of a Lean Daily Management System into an Antimicrobial Stewardship Program

**DOI:** 10.1097/pq9.0000000000000384

**Published:** 2021-03-10

**Authors:** Ann L. Wirtz, Elizabeth A. Monsees, Kate A. Gibbs, Angela L. Myers, Alaina N. Burns, Brian R. Lee, Rana E. El Feghaly, Gina M. Weddle, James C. Day, Amol V. Purandare, Jennifer L. Goldman

**Affiliations:** From the * Department of Pharmacy, Children’s Mercy Kansas City, University of Missouri, Kansas City, Mo.; † Patient Care Services Research, Children’s Mercy Kansas City, University of Missouri, Kansas City, Mo.; ‡ Division of Ophthalmology, Children’s Mercy Kansas City, University of Missouri, Kansas City, Mo.; § Division of Infectious Diseases, Children’s Mercy Kansas City, University of Missouri, Kansas City, Mo.; ¶ Department of Advanced Practice Programs, Children’s Mercy Kansas City, University of Missouri, Kansas City, Mo.

## Abstract

Growing demands and limited guidance on efficient use of resources to advance stewardship initiatives challenge antimicrobial stewardship programs (ASP).

**Methods:**

The primary aim was to incorporate a Lean Readiness and Metrics Board (RMB) into ASP and assess team member accountability and satisfaction with weekly 15-minute huddle participation within 1 year of implementation. ASP team survey data were analyzed for comments regarding Lean integration, team communication, and productivity. The second aim was to develop 5 shared metrics associated with quality, people, delivery, safety, and stewardship and evaluate ASP team productivity by assessing the impact of projects targeted at each specific metric. Pharmacist-physician ASP scheduling conflicts were addressed through identified rounding times under the “People” metric. The “Quality” metric assessed ASP intervention disagreement rate and collaborations that occurred to reduce disagreement. ASP tracked the number of individuals educated by ASP monthly through the “Delivery” metric.

**Results:**

Since August 2018, ASP replaced hour-long monthly meetings with weekly huddles at the RMB. On average, 14 members (88%) of the ASP participate weekly. Team members report improvement in communication and satisfaction with Lean integration. Metric utilization enhanced productivity. For the metrics under “People,” “Quality,” and “Delivery,” reduced scheduling conflicts occurred, the ASP intervention disagreement rate decreased (37.0%–25.6%; *P* < 0.001), and the ASP educated an average of 79 learners per month.

**Conclusions:**

Weekly huddles at the RMB enhanced communication and team accountability while visually displaying program needs, progress, and achievements. The RMB helps to ensure ongoing institutional commitment, and Lean methods show promise for evaluating and improving ASP productivity.

## INTRODUCTION

### Problem Description

Antimicrobial stewardship programs (ASP) have become essential to promote the appropriate use of antimicrobials.^1–3^ Recommendations from Infectious Diseases Society of America, The Joint Commission, and the Centers for Disease Control and Prevention (CDC) provide clear guidance on program requirements and implementation strategies to optimize antimicrobial therapy best.^1,2,4^ Traditional ASP metrics, such as antimicrobial days of therapy and rates of *Clostridioides difficile* infection, evaluate the clinical impact of ASPs on the improvement of antimicrobial use.^4^ These metrics are directly related to improving patient outcomes and are critical to evaluate ASP effectiveness.

Growing demands and limited guidance on how to efficiently use resources to advance stewardship initiatives challenge ASPs. Staffing dedicated to ASPs vary. Programs rely on a broad range of experts, including infectious diseases (ID) physicians, pharmacists, nurses, microbiologists, infection preventionists (IP), and data analysts.^5,6^ Expansion of services to the outpatient setting, education requirements, and additional clinical initiatives (ie, 48-hour timeout) further strain ASP resources. Despite the universal need to optimize time and assets, there is a lack of literature describing how ASPs should improve efficiency by fully engaging each member of the expert team and evaluating the corresponding impact on program productivity.

Since 2008, a hospital-wide ASP has reduced unnecessary antimicrobial use in a pediatric academic medical center through a prospective audit with feedback (PAF) stewardship.^7,8^ The ASP consists of a large, 16-member team incorporating ID physicians, pharmacists, microbiologists, a data analyst, an advanced practice provider (APP), and a nurse with IP expertise. Two physicians, 2 pharmacists, a nurse, an APP, and a data analyst have portions of full-time equivalents (FTEs) dedicated to ASP. All other team members volunteer time for ASP activities, including PAF reviews, education, quality improvement initiatives, and microbiology requests, depending on member position. Historically, all ASP team members met monthly; however, as the ASP expanded (ie, outpatient ASP, nursing initiatives), the team experienced ineffective communication, inability to quantify program needs, and a lack of shared accountability with monthly meetings. Despite success with traditional outcome metrics [eg, reducing antimicrobial days of therapy (DOT) per 1000 patient-days], ASP rarely assessed process metrics, such as program productivity with PAF stewardship and frequency of other ASP activities (ie, education).

Lean methodology, which originated from Toyota, focuses on increasing value-added and reducing unnecessary waste by streamlining workflow and improving problem-solving techniques, and establishing escalation strategies. Lean Daily Management System (DMS) is a system used to ensure all processes are fully functioning and problems are escalated promptly.^9,10^ Our hospital introduced Lean DMS, including tiered daily readiness huddles, in 2015. The ASP team identified an opportunity to integrate the hospital-wide Lean DMS approach into the ASP to explore if this method of continuous improvement would address the team’s concerns and promote the further assessment of program productivity.

### Specific Aims

The primary aim was to incorporate a Lean Readiness and Metrics Board (RMB) into ASP and assess team member accountability and satisfaction with weekly 15-minute huddle participation within 1 year of implementation. The second aim was to develop 5 shared metrics associated with quality, people, delivery, safety, and stewardship and evaluate ASP team productivity by assessing the impact of projects targeted at each specific metric.

## METHODS

### The Environment

This quality improvement (QI) project occurred in a 354-bed, free-standing, nonprofit academic pediatric medical center, which provides comprehensive primary and tertiary care in 40 pediatric subspecialties to a 5-state, 100-county region. There are approximately 15,000 admissions annually.

### Antimicrobial Management: Institutional

The ASP pharmacists, physicians, and APP at this institution have conducted over 35,000 PAF reviews for selected antimicrobials prescribed for 2 calendar days. On average, 40 PAF reviews result in an intervention each month. Outcome data, including DOT per 1000 patient days, PAF disposition (eg, antibiotic stopped, changed route, etc.), multidrug-resistant infection rates, and *Clostridioides difficile* infection rates, are evaluated monthly. Additionally, the CDC’s National Healthcare Surveillance Network and several national collaboratives benchmark internal antibiotic utilization data, facilitating subsequent QI initiatives. ASP shares data widely with hospital stakeholders, including individual clinical departments, Infection Prevention, Pharmacy and Therapeutics, and the Hospital Board.

### Lean Introduction and Integration

The interdisciplinary ASP team attended a 2-day, in-hospital Lean DMS workshop designed to produce the following deliverables: establish programmatic goals, identify weekly readiness components, develop and track prioritization metrics, create a visual display of activities, and communicate progress. The first day, the workshop leader introduced Lean DMS fundamental concepts, and the team customized RMB for an intended 15-minute huddle with all ASP team members. The second day, the ASP team presented the RMBs to hospital Lean experts and administrators for feedback.

**Fig. 1.**
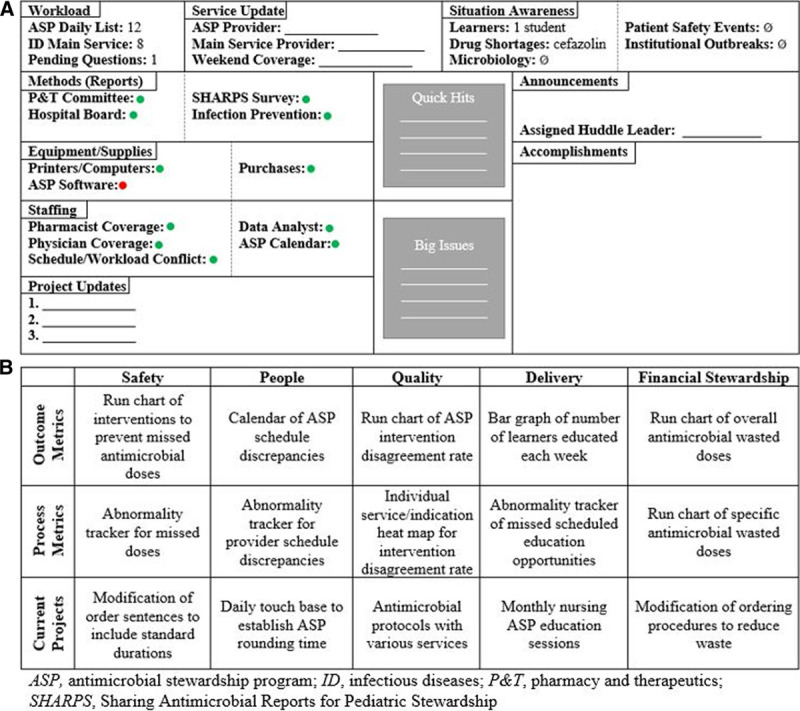
Overview of Lean daily management system. A, Readiness board. B, Program productivity metrics board. P&T, pharmacy and therapeutics; SHARPS, Sharing Antimicrobial Reports for Pediatric Stewardship.

Using the standardized hospital template, we established the RMB, which incorporated traditional ASP metrics and was customized to meet specific program needs. One intent of the RMB is to communicate ASP daily workload and scheduling across ASP and ID patient teams (Fig. 1A). A stoplight system tracks ASP team member availability, completion of recurring report requests, and equipment performance. Red represents delays or barriers to specific readiness variables, while green represents optimal performance. This visual approach also allows the ASP team to understand the team members’ availability to lead initiatives or if they are unavailable because of competing demands. In response, resources are prioritized to lend support to achieve project completion as team members share responsibility for collective enterprises under “Project Updates.” The “Situational Awareness” section promotes discussion of drug shortages, microbiology issues, and patient safety events. All team members add identified challenges to “Quick Hits” (internal problems solved within 72 hours) or “Big Issues” (program challenges requiring additional resources or collaboration with other departments to resolve). Dissemination of recognitions, reminders, and contributions occurs during “Announcements” and “Acknowledgements.” The ASP pharmacists and data analyst are primarily responsible for updating the RMB each week, although all team members are encouraged to add content.

The RMB also displays ASP measures selected to evaluate program productivity while incorporating Lean fundamentals to reduce waste, improve satisfaction, and optimize workflow processes (Fig. 1B). Metrics serve as visual depictions of progress to-date and promote discussions for the allocation of resources. Metrics were selected based on hospital-wide prioritized categories: safety, people, quality, delivery, and financial Stewardship. Each DMS category is associated with measures of process performance, desired outcomes, and improvement projects unique to the practice area. Measures and projects are regularly evaluated for impact and updated according to program needs. ASP team members verbalized opportunities for improvement during group discussion, which resulted in initial metrics development. Minimal baseline data collection existed for the metrics initially chosen before the introduction of Lean.

**Fig. 2.**
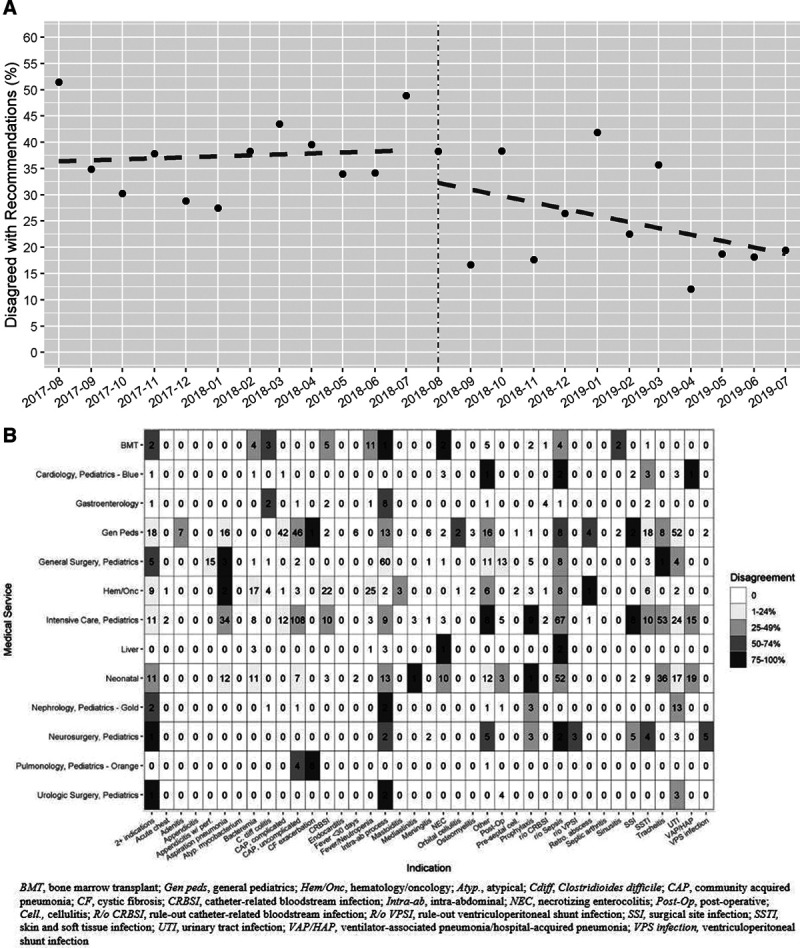
Quality metric data. A, Disagreement by month. Dashed line indicates the linear slope for the disagreement rate. B, Disagreement by specialty and clinical indication. BMT, bone marrow transplantation; Gen peds, general pediatrics; Hem/Onc, hematology/oncology; Atyp., atypical; Cdiff, *Clostridioides difficile*; CAP, community-acquired pneumonia; CF, cystic fibrosis; CRBSI, catheter-related bloodstream infection; Intra-ab, intra-abdominal; NEC, necrotizing enterocolitis; Post-Op, postoperative; Cell, cellulitis; R/o CRBSI, rule-out catheter-related bloodstream infection; R/o VPSI, rule-out ventriculoperitoneal shunt infection; SSI, surgical site infection; SSTI, skin and soft tissue infection; UTI, urinary tract infection; VAP/HAP, ventilator-associated pneumonia/hospital-acquired pneumonia; VPS infection, ventriculoperitoneal shunt infection.

Under “People,” the team chose to address schedule discrepancies between team members performing daily ASP rounds. Pharmacists reported a delay in stewardship rounds due to ID physician availability resulting in wasted time. A calendar and abnormality tracker were completed by ASP pharmacists daily to identify frequency and reason for discrepancies with a performance target of zero discrepancies per month. As an intervention, the ASP pharmacist and physician established rounding times based on each day’s schedule at the beginning of the week to decrease conflicts and improve time to ASP rounds. For “Quality,” the team tracked the disagreement rate with ASP interventions to prescribers through PAF stewardship to determine how the team could continue to work collaboratively with other teams receiving ASP interventions. The rate of disagreement serves as a proxy for analyzing customer satisfaction and provider agreement (ie, satisfaction) with ASP. Disagreement rate was obtained through ASP documentation in a preexisting ASP repository when a provider disagreed with both the primary and compromise intervention. The data analyst prepared a segmented regression model of the disagreement rate and heat maps displaying individual medical services and intervention types weekly, allowing team members to prioritize and tailor interventions to reduce disagreements (Fig. 2A, B). ASP team members elected to monitor each month’s disagreement rate and intervene based on team discussion of the data trends. The team initially identified medical services with higher rates of disagreement, utilizing heat maps, and identified opportunities for collaboration (Fig. 2B). For this article, we used Pearson’s χ^2^ test to compare disagreement rates before and after Lean implementation. A segmented general linear regression model was run to depict the changes in disagreement trends.

For “Delivery,” the team elected to track the number of learners educated by ASP as the team had no prior established methods to prioritize education and quantify educational reach. To optimize educational efforts, ASP set a goal of reaching at least 50 learners per month. Learners included individuals at any level of training in any profession receiving ASP education in a formal setting. Individual ASP team members counted the number of learners at each educational activity, and pharmacists monitored the number of learners per week on a bar graph. An abnormality tracker identified reasons for missed scheduled educational opportunities by ASP team members.

Rather than daily huddles, ASP opted to implement once weekly, 15-minute huddles at the RMB. A rotating ASP team member leads huddles, and other team members, either physically present or on the phone, provide project updates and participate in the discussion as needed. To ensure brevity, after 3 communication exchanges on a single topic, the issue is tabled. A small group is designated to discuss the topic further and return to the next huddle with follow-up.

One year following the Lean implementation in preparation for a strategic planning session, ASP team members participated in a survey to assess team strengths, weaknesses, and opportunities for further development. Together, the ASP team reviewed survey data for trends from comments regarding Lean integration, team communication, and productivity.

## RESULTS

### Team Member Accountability

Since August 2018, the ASP has replaced hour-long monthly meetings with weekly, 15-minute huddles at the RMB. On average, 14 members (88%) of the ASP team attend each weekly huddle. All ASP team members have led huddle discussions. Additionally, all have participated in the problem-solving of “Quick Hits” and “Big Issues.” For example, the report utilized for PAF stewardship was malfunctioning. It was added as a “Big Issue.” Progress on this was assessed each week at huddle; however, when the issue was not resolved timely, it was promptly escalated and resolved.

The RMB huddle has provided opportunities for weekly updates from collaborating departments, including microbiology and IP. An individual representing the departments is encouraged to serve as project lead. Discussion of recent infections with carbapenem-resistant organisms has led to ASP/IP collaborations, including a thorough review of antibiotic prescribing by the ASP pharmacist to determine the impact of antibiotic prescription on organism development.

### Productivity Metrics

Individual projects were implemented, targeting each productivity metric described. For “People,” identified established rounding times resulted in a decrease in ASP schedule conflicts. Following this intervention, no greater than one schedule discrepancy occurred each month. Most months had no discrepancies; therefore, this metric was replaced based on program needs within five months following Lean integration. Using the “Quality” metric, the team focused on collaborations with specific services to decrease the disagreement rate. Dedicated, intentional collaborations with surgical subspecialties, for instance, led to the development of standardized protocols for the management of specific surgical site infections. Before Lean implementation, the proportion of ASP interventions the provider disagreed with was 37%; however, following the implementation of Lean, the disagreement percentage decreased to 25.6% (*P* < 0.001; Fig. 2B). ASP continues to focus on this metric and design projects to reduce the disagreement rate further. For “Delivery,” ASP reached 476 learners within the first 6 months, with an average of 79 learners per month and few missed scheduled educational opportunities. This metric was also replaced to focus on program needs.

### Survey

Within the ASP team member survey, 8 of the direct ASP team members entered comments that identified Lean as a specific team strength. As one member reported,

The multidisciplinary nature of the team (physicians, pharmacists, nurse practitioner, RN, data-analyst/epidemiology) provides us with a unique opportunity to be able to approach services and problem solving from many different perspectives. This diversity helps us to strive to be more inclusive of all providers/patients/family members in our approach to ASP. I also think the strong connection with education and empowering providers to be more confident in their antimicrobial knowledge and guideline recommendations is another great aspect of ASP. We are not the antimicrobial police but instead a dedicated team to help education/support frontline providers to ensure patients are getting the most optimal antimicrobial therapy.

ASP team members described communication as getting “better with the lean huddle approach.” Goals and opportunities for the next 3 years include development into “hospital Lean-leaders.”

## DISCUSSION

The team implemented Lean DMS with the RMB to address the initial concerns surrounding meeting structure and communication, resource needs, and productivity. To the authors’ knowledge, this is the first description of the integration of Lean DMS into ASP efforts, resulting in a novel approach for practical ongoing stewardship program assessment. Lean methodology is well-described within healthcare settings, with a primary focus of optimizing performance, eliminating unnecessary waste, and increasing patient satisfaction.^9–11^ These fundamental values are beneficial for ASPs, which must capitalize on productivity to meet growing regulatory requirements.^1–3^

As described, the ASP team at our institution is robust and consists of multiple disciplines. Most team members have competing priorities (eg, clinical service time, research), limiting the available time to engage in the ASP. Division of the hour-long monthly meeting into 15-minute weekly huddles at the RMB was marketable to team members because of meeting brevity. Problem-solving and project updates now occur weekly, which increases time to recognize and escalate unresolved issues and provides all team members multiple opportunities to participate each month. An initial barrier was identifying a huddle time to optimize member attendance. ASP selected a 15-minute window immediately after weekly ID didactic sessions, which most team members attend. As demonstrated by the high attendance rate, huddle participation, and survey results, this communication method has engaged and satisfied team members. While other institutional ASPs may consist of fewer members, national recommendations continue to highlight opportunities for engaging various disciplines (ie, nursing, microbiology) in ASP efforts.^1,12^ The RMB serves as a viable option to engage a large multidisciplinary group and provide a platform for microbiology and IP to participate in the ASP.

The development of program evaluation metrics was essential to monitor productivity and thoroughly assess program function. Antibiotic DOT per 1000 patient days continues to serve as a necessary metric for evaluating ASP activity. Our team observed a decrease in overall antibiotic DOT per 1000 patient days; however, we cannot directly attribute this to incorporation of Lean methodology because of additional initiatives implanted during this time. The described metrics aimed to improve program productivity by improving processes, enhancing satisfaction, and reducing waste. Team discussion during metric selection was enlightening as team members identified several novel areas of improvement, which were not prior focuses of the program such as education of learners. Reduction in scheduling conflicts with the “People” metric streamlined processes to decrease wasted time, which was necessary to expand ASP services to the outpatient setting and prioritize other improvement activities. With the “Quality” metric, the ASP recognized that while our antibiotic utilization data were comparable with peer institutions, there were still many opportunities to improve satisfaction with ASP services by reducing the disagreement rate. Additionally, several metrics coincide with data necessary for current accreditation standards. For example, the “Delivery” metric highlighted the immense amount of education being performed each month, which served as a quantifiable example of how the program met ASP education requirements.

The integration of Lean RMB into ASP allowed team members to become more familiar with QI and problem-solving methods when considering the implementation and dissemination of ASP interventions.^13^ An initial challenge included adapting Lean processes and understanding terminology. Many team members were new to Lean fundamentals; however, the initial workshop, assistance from a QI mentor, and continued team member engagement in huddle increased familiarity. Additionally, the RMB serves as a visual display of current efforts, programmatic successes, and challenges. As numerous departments within the institution engage in Lean DMS, the RMB is a familiar and informative tool for the hospital’s administrators. By displaying ASP goals and progress, the RMB helps to ensure ongoing institutional commitment, and Lean methods show promise for evaluating and improving ASP productivity.

### Limitations

Before Lean integration, no formal tracking mechanisms existed for data including attendance at monthly meetings, frequency of scheduling conflicts with ASP reviews, and the number of learners educated. This deficiency prohibits a formal comparison; however, it highlights the benefit of the RMB as these data are now collected. A potential limitation of the ASP RMB for other institutions is the requirement of systematic data collection and analysis to display meaningful information, to demonstrate progress, and to stimulate positive ideas for change. Given this necessary time commitment, ASPs with limited resources may struggle to implement multiple metrics concurrently. At our institution, updating the RMB and metrics added workload to the pharmacists’ role, which is limited due to competing priorities (ie, rounding). As metrics are updated, we now assign the responsibility of tracking metric data to specific team members to increase shared workload. Metrics that were meaningful and impactful to our ASP may not have the same impact at other institutions based on program activities. Additionally, selecting meaningful metrics is challenging, including within our ASP, as it can be time-consuming and taxing to continue identifying thoughtful metrics.

### Next Steps

The integration of Lean methodology into ASPs requires ongoing team commitment to stay current with changing hospital and ASP needs. The sustainability of Lean is an area for further research for this program. A situation-target-proposal process has subsequently been developed by the ASP to standardize the group’s collaborative approach for identifying, evaluating, and addressing situations. The situation-target-proposal method will need further assessment for integration into weekly huddles.

## CONCLUDING SUMMARY

By applying Lean methodology into an existing ASP, the team created an inclusive approach that improved communication and productivity within a large multidisciplinary team. Combined with traditional ASP metrics, the development of internal measures has aided in the reduction of waste, increased satisfaction, and optimization of ASP activities. It is affirming that a year after the implementation of Lean, members reported a sense of accomplishment and heightened collaboration. As such, the authors believe this approach has utility for replication among ASPs interested in understanding how best to assess team productivity and performance.

## DISCLOSURE

The authors have no financial interest to declare in relation to the content of this article.

## Acknowledgments

The authors acknowledge Tom Belt. He led the development sessions and served as a beneficial resource and the Children’s Mercy administration for support of Lean DMS training for the ASP group.
